# Heterotopic pancreatic tissue in the gastric antrum an incidental finding during bariatric surgery: A case report and literature review

**DOI:** 10.1016/j.ijscr.2019.12.040

**Published:** 2020-01-09

**Authors:** Awadh Alqahtani, Emad Aljohani, Fahad Almadi, Srikar Billa, Mohammad Alqahtani, Hisham Alkhaldi

**Affiliations:** a King Saud University, Riyadh, Saudi Arabia; b Department of Surgery, College of Medicine, Prince Sattam Bin Abdulaziz University, Al-kharj, Saudi Arabia; c King Abdulaziz Medical City, Riyadh, Saudi Arabia; d Dr.Suliman Alhabib Hospital, Riyadh, Saudi Arabia; e King Khalid University, Abha, Saudi Arabia; f Histopathology Department, Dr.Suliman Alhabib Hospital, Riyadh, Saudi Arabia

**Keywords:** Heterotopic pancreas, Ectopic pancreas, Laproscopic sleeve gastrectomy, Mini gastric bypass

## Abstract

**Introduction:**

Heterotopic pancreas, also known as ectopic pancreas, is defined as the presence of pancreatic tissue outside its normal location and without anatomic and vascular continuity with the main body of the pancreas. The incidence of heterotopic pancreas has been reported as 0.5 % during laparotomies and at autopsy ranging from 0.6–14 %.

**Presentation of case:**

A 28 years old obese male, medically free electively admitted for laparoscopic sleeve gastrectomy. Intraoperatively after dissecting the greater omentum from the stomach and inserting the 36 Fr gastric bougie, small oval shape mass about 1 cm close to the lesser curvature on the anterior surface of the gastric antrum. Macroscopically benign looking and a thorough laparoscopic exploration showed no signs of other organs involvement. Antrectomy and mini gastric bypass done. The postoperative course was uneventful. The histopathological examination of the antrectomy specimen showed heterotopic pancreas in the subserosa of gastric antrum.

**Discussion:**

The Heterotopic pancreatic tissue can be discovered in the stomach (particularly antrum), duodenum, jejunum, or a Meckel diverticulum. Surgical resection is the mainstay treatment if the heterotopic pancreas is symptomatic or when the lesion is found incidentally during surgery in order to prevent complications.

**Conclusion:**

Heterotopic pancreas should always be considered in the differential diagnosis of incidentally found gastric lesions and can be safely resected. This is the first case report of gastric heterotopic pancreas which is found incidentally during a bariatric surgery procedure.

## Highlights

• Heterotopic pancreas, is presence of pancreatic tissue outside its normal location without anatomic continuity with the main body of the pancreas.
• The Heterotopic pancreas can be in the stomach, duodenum, jejunum, or a Meckel diverticulum.
• This is the first reported case of gastric heterotopic pancreas found during a bariatric surgery procedure.

## 1 Introduction

Heterotopic pancreas, also known as ectopic pancreas, accessory or aberrant pancreas is defined as the presence of pancreatic tissue outside its normal location and without anatomic and vascular continuity with the main body of the pancreas [1]. The incidence of heterotopic pancreas has been reported as 0.5 % during laparotomies and at autopsy ranging from 0.6–14 % [2]. Most patients are asymptomatic and typically incidentally discovered while undergoing laparotomy for other indications or endoscopic examinations of the gastrointestinal tract or at autopsy. At pathology, the gross appearance of a typical heterotopic pancreas in the stomach is a firm, round or oval subepithelial lesion and the presence of a characteristic central dimpling or umblication that is due to the opening of the duct [3]. We are reporting the first case report of gastric heterotopic pancreas which is found incidentally during a bariatric surgery procedure, this work has been reported in line with the SCARE criteria [4].

## 2 Presentation of case

**Fig. 1:**
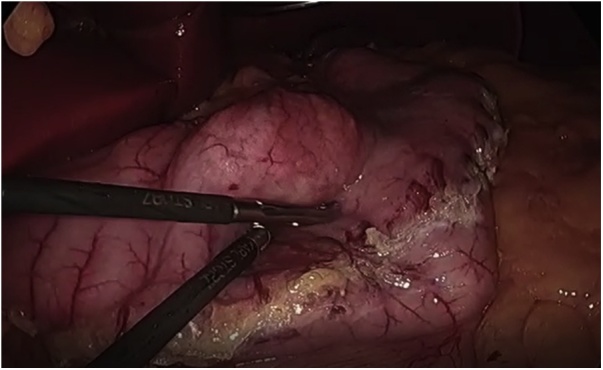
laparoscopic view of lesser curvature mass.

**Fig. 2:**
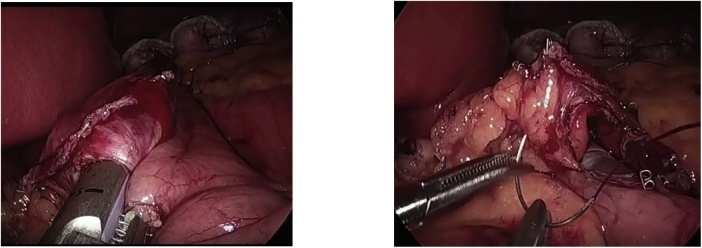
antrectomy and mini gastric bypass.

**Fig. 3:**
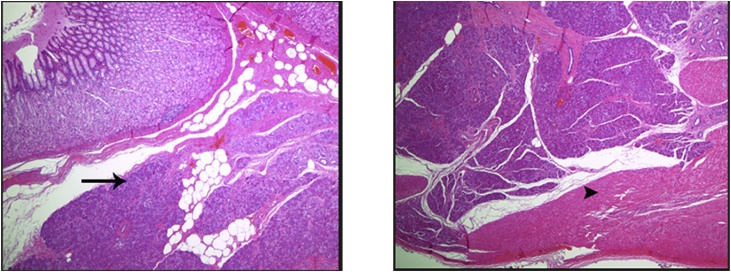
The resected stomach body wall, shows heterotopic pancreatic tissue that extends from the submucosa layer (arrow) to the subserosal layer within the muscularis propria (arrow head).

**Fig. 4:**
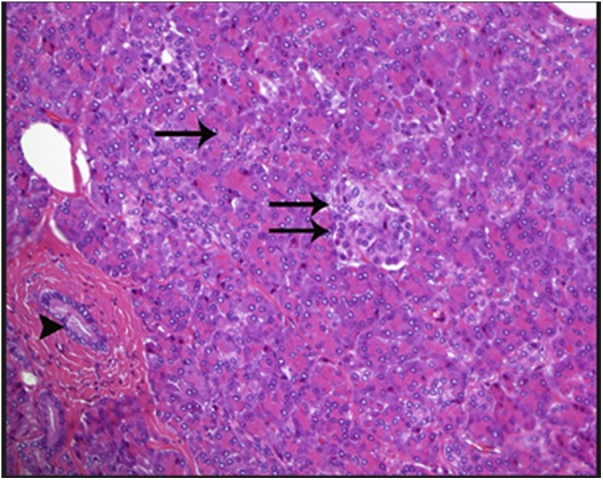
The heterotopic pancreatic tissue shows features of "total" hetetropia, including pancreatic ducts (arrow head) and exocrine glands (acinar cells, single arrow) and endocrine glands (islet cells, double arrow).

A 28 years old obese male with BMI 46 presented to our institute, asking about the proper bariatric surgery for his morbid obesity. After a thorough history, physical examination and laboratory investigations, he was booked for laparoscopic sleeve gastrectomy. Intraoperatively after dividing the greater omentum from the stomach and inserting the 36 Fr gastric bougie, we noticed a small about 1 cm oval shaped mass close to the lesser curvature on the anterior surface of the gastric antrum. (Fig. 1). Macroscopically appeared benign and a thorough laparoscopic exploration done and found no signs of malignancy on examination. So, a decision to do antrectomy and mini gastric bypass was taken (Fig. 2). The post-operative course was uneventful, discharged home by the second post-operative day. Two weeks after the discharge, the histopathology results of the antrectomy specimen showed the mass is suggestive of heterotopic pancreas in the subserosa of gastric antrum. The histopathological examination showed a mass that extends from the submucosal to the subserosal areas of the segment excised (Fig. 3). Microscopically, the mass was composed of total pancreatic heterotopia (Fig. 4). The elements noted include predominantly exocrine (acinar) pancreatic tissue, scattered pancreatic ducts and scattered endocrine (islets). The patient followed at 3 months, 6 months post operatively and was loosing acceptable weight to these time periods.

## 3 Discussion

Heterotopic pancreatic tissue is an aberrant focus of normally developed pancreatic tissue that lacks anatomic and vascular continuity with the main organ and can be found in various locations. Most Heterotopic pancreatic tissue is discovered in the stomach (particularly antrum), duodenum, jejunum, or a Meckel diverticulum. Other locations include the ileum, liver, spleen, biliary tract, mesentery, fallopian tubes, or umbilicus. Heterotopic pancreatic tissue is mostly located in the submucosa but in some instances, it can be found in the muscularis or serosa [4].

Heterotopic pancreas is usually found incidentally and is generally asymptomatic, but it may become clinically evident depending on its size, anatomical location and the pathological changes similar to orthotopic pancreas, particularly cystic degeneration, ectopic pancreatitis and even malignant degeneration [5]. Symptoms can include nausea, vomiting, epigastric pain, dyspepsia, abdominal fullness, and melena. The most common symptom is epigastric pain. About a third of symptomatic patients report clinical symptoms that mimic disease related to the organ in which the tissue resides [6].

Although the diagnosis of heterotopic pancreas is difficult preoperatively, there are few characteristic radiographic and endoscopic features that helps in its identification. Barium studies of gastric heterotopic pancreas can be seen as a rounded filling defect with a central indentation. Contrast enhanced computed tomography can sometimes demonstrate nondiagnostic findings such as exophytic bowel wall lesions or mural wall thickening [7]. Heterotopic pancreas of the stomach and duodenum has characteristic CT findings that differ from those of gastric submucosal tumors such as gastrointestinal stromal tumor (GIST) and Leiomyoma. Five criteria on CT have been used with good sensitivity and specificity to help differentiate between ectopic pancreas and GIST. These criteria are as follows: pre-pyloric antrum or duodenum in location, an ill-defined border, an endoluminal growth pattern, a long diameter/short diameter ratio of greater than 1.4, and prominent mucosal enhancement. When two or more criteria are met, the sensitivity and specificity for diagnosing ectopic pancreas approaches 100 % and 82.5 %, respectively [8]. However, Definitive diagnosis of ectopic pancreas is always made histologically [9].

Surgical resection is the mainstay treatment if the heterotopic pancreas in symptomatic or when the lesion is found incidentally during surgery in order to prevent complications. It can be resected open or laparoscopically or endoscopically

## 4 Conclusion

Heterotopic pancreas should always be considered in the differential diagnosis of incidentally found gastric lesions and can be safely resected. This is the first reported case of gastric heterotopic pancreas which is found incidentally during a bariatric surgery procedure in a morbidly obese patient which changed the decision of doing sleeve gastrectomy to mini gastric bypass.

## Sources of funding

No fund to my research to be disclosed.

## Ethical approval

This is case report study and ethical approval not required.

## Consent

The patient himself signed the consent and No characteristics are altered in my study.

## Author contribution

Dr.Awadh Alqahtani (literature review).

Dr.Emad Aljohani (case description and discussion).

Dr.Fahad Almadi (collected the images from the patient file).

Dr.srikar billa (collected the patient history and examination from the file and he wrote the references).

Dr.Mohammad Alqahtani (literature review).

Dr.Hisham Alkhaldi (review the pathology slides).

## Registration of research studies

NA.

## Guarantor

Dr.Awadh Alqahtani.

## Provenance and peer review

Not commissioned, externally peer-reviewed.

## Declaration of Competing Interest

No conflict of interest.
